# The spatial effects of the household's food insecurity levels in Ethiopia: by ordinal geo-additive model

**DOI:** 10.3389/fnut.2024.1330822

**Published:** 2024-02-29

**Authors:** Habtamu T. Wubetie, Temesgen Zewotir, Aweke A. Mitku, Zelalem G. Dessie

**Affiliations:** ^1^College of Science, Bahir Dar University, Bahir Dar, Ethiopia; ^2^Department of Statistics, College of Natural and Computational Science, University of Gondar, Gondar, Ethiopia; ^3^School of Mathematics, Statistics and Computer Science, University of KwaZulu-Natal, Durban, South Africa

**Keywords:** panel data, Markov random field, tensor product, unobserved heterogeneity, spatial effect

## Abstract

**Background:**

Food insecurity and vulnerability in Ethiopia are historical problems due to natural- and human-made disasters, which affect a wide range of areas at a higher magnitude with adverse effects on the overall health of households. In Ethiopia, the problem is wider with higher magnitude. Moreover, this geographical distribution of this challenge remains unexplored regarding the effects of cultures and shocks, despite previous case studies suggesting the effects of shocks and other factors. Hence, this study aims to assess the geographic distribution of corrected-food insecurity levels (FCSL) across zones and explore the comprehensive effects of diverse factors on each level of a household's food insecurity.

**Method:**

This study analyzes three-term household-based panel data for years 2012, 2014, and 2016 with a total sample size of 11505 covering the all regional states of the country. An extended additive model, with empirical Bayes estimation by modeling both structured spatial effects using Markov random field or tensor product and unstructured effects using Gaussian, was adopted to assess the spatial distribution of FCSL across zones and to further explore the comprehensive effect of geographic, environmental, and socioeconomic factors on the locally adjusted measure.

**Result:**

Despite a chronological decline, a substantial portion of Ethiopian households remains food insecure (25%) and vulnerable (27.08%). The Markov random field (MRF) model is the best fit based on GVC, revealing that 90.04% of the total variation is explained by the spatial effects. Most of the northern and south-western areas and south-east and north-west areas are hot spot zones of food insecurity and vulnerability in the country. Moreover, factors such as education, urbanization, having a job, fertilizer usage in cropping, sanitation, and farming livestock and crops have a significant influence on reducing a household's probability of being at higher food insecurity levels (insecurity and vulnerability), whereas shocks occurrence and small land size ownership have worsened it.

**Conclusion:**

Chronically food insecure zones showed a strong cluster in the northern and south-western areas of the country, even though higher levels of household food insecurity in Ethiopia have shown a declining trend over the years. Therefore, in these areas, interventions addressing spatial structure factors, particularly urbanization, education, early marriage control, and job creation, along with controlling conflict and drought effect by food aid and selected coping strategies, and performing integrated farming by conserving land and the environment of zones can help to reduce a household's probability of being at higher food insecurity levels.

## Introduction

The Food and Agriculture Organization (FAO) of the United Nations defined that “food security is achieved when all people, at all times, have physical and economic access to sufficient, safe, and nutritious food to meet their dietary needs and food preferences for an active and healthy life”(1). Households with lower nutritious food intake compared to food secured households are related to food insecure and vulnerable households (1). The adverse effects of food insecurity on mental health, increased risk of being stunted, wasted, and obesity on children, and having type 2 diabetes (2, 3) are typical. The impact of food insecurity also extended to low human development, leading to higher poverty and inequality (4), along with low immunity and less productivity (5–7).

Recent global shocks, notably the US–China economic trade war, the global pandemic “COVID19” (3, 8–12), and the Russia–Ukraine (9, 13) war, have increased the risk of the food security of households in every country worldwide (14). In addition to the gradual decline in food security since 2014 (15), these shocks resulted in 928 million food insecure people in 2020, an increase of 148 million compared to 2019 (15). Furthermore, there are 122 million more hungry people in 2022 than before the COVID-19 pandemic (16) and 3.4 million additional stunting cases in 2022 due to COVID19. The global hunger range is between 702 and 828 million in 2021 (3). As close to the main driver factor, the higher proportion of food insecure reside in countries exhibits conflicts and wars (15). This region also contributes 80% of the global stunted population (2, 15). Moreover, the chronologically declined global severe food insecurity prevalence and other malnutrition problems (2) are inflated mainly after COVID-19 pandemic (3, 15, 17), and the estimated increase in food insecure people population in 2020 is equal to that of the previous 5 years combined (3, 15).

Primarily, the developing countries, such as African (52%), Asian (34%), and Latin American (9%), cover larger proportion of global food insecure population (2) mainly due to climate change (15, 18). In addition to vast intra and extra country conflicts in Africa, the frequent occurrence of drought in the region has been the major cause of crop and livestock losses (89%) (19), which leads the continent to take a larger proportion of severe food insecure population (2). The prevalence of food insecurity is high (42%) in East Africa (20), especially Ethiopia faced food insecurity for many years; from 1979/80 and 1995/96 (21), in recent years (22) especially from 2019 to 2021 more than half (56%) of the total population suffered from moderate or severe food insecurity (23).

Currently, the bottleneck problem of Ethiopia is “the poor and large population.” In Ethiopia, 68% of the population faced multidimensional poverty (24) and the population is over 117 million in 2021 (109). Among them, 78% resides in rural areas, relying on traditional farming methods that are prone to climate change (25). The influence of global shocks, such as global pandemic “COVID19,” US–China economic trade war, and Russia–Ukraine war, and the internal conflicts (primarily on the Norther and Western Ethiopia) leads millions of people to relocate (110) and frequent droughts (111) and collectively aggravates the food insecurity. These circumstances lead to more than 20 million people experiencing hunger and requiring rapid aid from World Food Program (WFP), FAO, and other aid organizations (109).

Several studies conducted in the country indicated that conflict and drought factors, such as land size, dependency ratio, credit services, family size, household assets, number of livestock, fertilizer usage, employment, household head age, sex, education, and social protection program (26, 27), can affect the household's food security (28–34). For food insecure countries, including Ethiopia, with the advantage of the two greatest potential resources, i.e., “the people and the productive land and water,” efficient usage of these resources by investing both people and productivity can mitigate the food security problem (1, 35, 36). In addition to the numerous investigation results on the country's food insecurity, Ethiopia is a traditional country where the cultural ceremonies and religious events bring a different feeding pattern to the usual feeding culture. The food insecurity is not yet evaluated using the recommended locally adopted cut-points for the food consumption score (FCS) measure of WFP, considering that the local factors effect resulted in a divers feeding pattern (e.g., exclusion of small amounts of food items from the diet during measurement extends to include sugar and oil consumption) (37, 38). Furthermore, very few studies in the country evaluated the non-linear effects of the covariates (22, 39) without considering the spatial effects. Moreover, even though similar comprehensive global studies, such as spatial correlation of food security in developing countries (18) and in China (39), have been conducted, the effect of local factors on food insecurity level cut-point is not considered.

Therefore, in this study, to give a deeper insight, we hypothesize that neighboring zones are more likely in the food security level than any other zones, and we need to assess the comprehensive linear and non-linear effects of geographic, environmental, and socioeconomic factors on each corrected levels of household's food insecurity. The study aims to provide comprehensive perspective, helping us to clearly understand the distribution nature of the household's food insecurity levels across administrative zones and identify key determinants among comprehensive factors influencing households at different levels of food insecurity dynamics. This information enable us to cluster the hot spot zones of food insecurity and vulnerability for area-specific mitigation using key factors.

We can apply the statistical models those have been widely used in research on the dynamics of households with food insecurity. In modeling such phenomena, the non-linear regression model, such as structured additive regression (STAR) models, alleviates the restriction that the model coefficients are constant in both linear and non-linear effects for different parts (quantiles or categories) of the distribution of y (given x), extending to include the geographical effects (40–47). In the Bayesian perspective, the empirical Bayes (EB) is preferred to full Bayes (FB) when achieving convergence of MCMC (Markov chain Monte Carlo) samples becomes a challenge. This choice is influenced by its fast optimization and unbiased estimation of the variance parameters, i.e., geo-additive models (48–52). Therefore, a novel model, i.e., the empirical Bayesian geo-additive model, by Augustin et al. (53) has been applied in this study.

From the exploratory analysis of the response variable, we observed the possible non-linear effect of continuous covariates across the response category, varying across administrative zone effects. This study aims to assess the distribution nature of the household's food insecurity levels across the administrative zones and identify key determinants from geographic, environmental, and socioeconomic factors, which contribute to different levels of locally adjusted food insecurity measures, using the ordinal geo-additive mixed model.

The remaining part of the paper is organized as follows: The method section incorporates the data sources, explanatory variables and the response considered in the analysis, and the software and the models employed; the result section interprets the main and significant outputs from descriptive and inferential statistical methods; the discussion section includes the result with respect to the existing literature; and finally, the conclusion section presents the main conclusions from the empirical result and correspondingly points out relevant interventions for policy implementation.

## Methods

### Data

**Figure 1:**
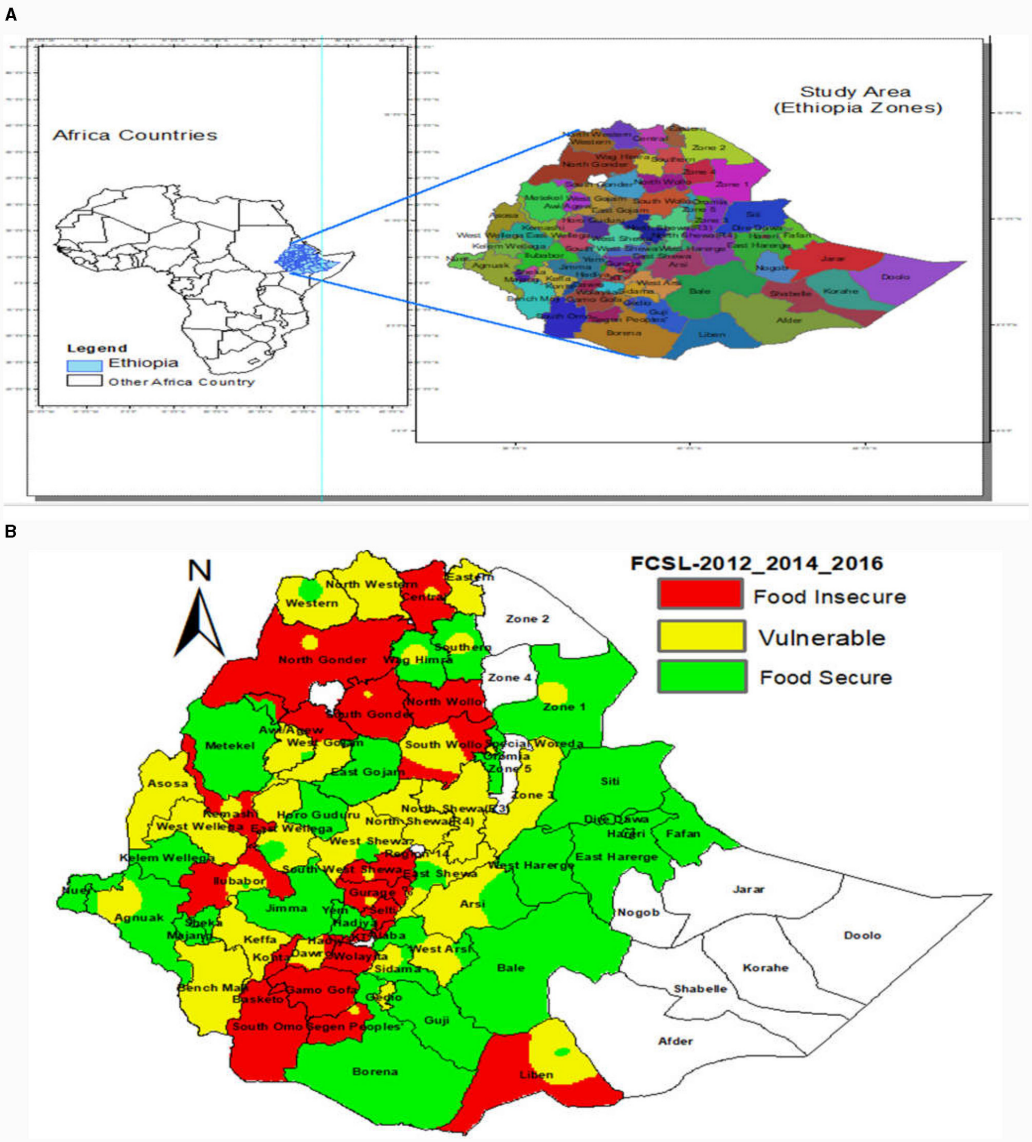
**(A)** Map of study Area or administrative zones of Ethiopia. **(B)** Observed spatial distribution of food insecurity levels based on average longitudinal data of years 2012, 2014, and 2016.

This study analyzed a total sample size of 11505 households (the three-term household-based panel data for years 2012, 2014, and 2016), which covers the whole regions of the country given by the study area plot as shown in Figure 1A. This study comprises a total sample size of 3,835 households, obtained through two-stage probability sampling across 64 administrative zones with three replications (enumeration areas and households). After removing, two times, longitudinally missed values (a household has only surveyed once in survey years), missing values are imputed or treated by longitudinal mean for longitudinally missed values in the response or explanatory variables and by mean imputation for households with missing values in some explanatory variables. In the first stage of sampling, 333 enumeration areas (290 rural and 43 urban) were selected using simple random sampling from the sample of the Annual Agricultural Sample Survey (AgSS) enumeration areas (EAs). The AgSS EAs were selected based on probability proportional to size of the total EAs in each region. This approach considers the population size of the populous regions for sufficient representation (Amhara, Oromiya, SNNP, and Tigray) even though some under representation has been observed for unpopulous regions (Afar, Benshangul Gumuz, Dire Dawa, Gambella, Harari, and Somalie regions). The second stage is selecting 3,969 households by simple random sampling from the enumeration areas with a response rate of 99.3%. The survey was designed to be implemented in a three-round timeframe based on the AgSS field schedule—the 1st round from September to October in 2011, 2013, and 2015; the 2nd round from November to December in 2011, 2013, and 2015; and 3rd round from January to April in 2012, 2014, and 2016. The data from households were collected using both paper-assisted personal interview (PAPI) and computer-assisted personal interview (CAPI) methods with minimal problems encountered such as limited electricity availability for re-charging the CAPI tablets during the fieldwork. The data source is the Ethiopian Socioeconomic Survey (ESS) of the World Bank data set, which is the first panel data in Ethiopia collected using a project of World Bank and CSA of Ethiopia that quantify household-level food security and related factors in rural and urban (small and medium towns) areas.

### Variable and measurements

The food security measurement is an ongoing problem, with various studies proposing and employing different measurement scales. This aspect includes a continuous, three-class ordinal scale or binary classification based on food consumption score (FCS) (1, 29, 54, 55). For more informative assessments and applications of policy interventions, instated of continuous and dichotomized response as food secure and insecure, this study applied ordinal food insecurity levels (FCSL), i.e., food-secure, vulnerable to food insecure, and food-insecure household (1, 56). In addition to the WFP's standard food consumption scale formulated based on 7 of days household food intake with cut points 21 and 35, to classify as food insecure, vulnerable, and secured; based on society dieting culture and pattern different cut point are recommended such as WFP recommend 28 and 42 for community sugar and oil consumption is 5 to 7 days a week (57), for Jordan households the FCS cut points used are 45 and 61 (58), and Baumann et al. (59) used 32 and 43 as cut points by excluding small amounts of food items.

As a traditional, historical, and religious country, Ethiopia has very large diet diversity in addition to various condiments and small amount of foods consumed during cultural ceremony and religious events. Hence, the response variable of this study is the weekly household's FCS of WFP with thresholds corrected for different feeding cultures of the society (59–61), using an effect-driven quantile clustering approach. This approach groups the food security score into a three-ordered class with cut-points of 35.5 and 49 to classify as food insecure, vulnerable to insecurity, and food secured (56).

The explanatory variables incorporated for analysis following dimension reduction, variable selection, and exploratory analysis on 91 original variables from ESS data set are eight categorical factors and 14 continuous covariates. In addition, the variables are assumed to have both linear and non-linear effects on food security levels. However, the principal component analysis by considering the Eigenvalue (>1), the proportion of variance explained from the total variance, and the subjective meaning of highly contributing components (62, 63) were used to reduces the dimension of geographic variables from 19 to 6 components explaining 76.12%, similarly 12 agricultural variables combined into 4 components explaining 53%, and 47 assets variables merged into 12 components explaining 50.11% of the total variance.

### Model

The food insecurity level (FCSL_i_) is a categorized version of a latent continuous variable of the original food consumption score, say *D*_*i*_ which is truncated normal, *D*_*i*_ = η_*i*_+ϵ_*i*_, where η_i_ is a predictor depending on covariates and parameters, and ϵ_i_ is an iid nx1 error vector, for I = 1, 2, …, 11505. The two variables, FCSL_i_ and *D*_*i*_, are linked by FCSL_i_= r if and only θ_*r*−1_ < *D*_*i*_ ≤ θ_*r*_, r =1, 2, 3, with an ordered threshold −∞ = θ_0_ < θ_1_ < θ_2_ < θ_3_ = ∞, for θ_1_ = 35.5 and θ_2_ = 49. Since the categorical response is a categorized version of a continuous latent response, the proportional-odds cumulative-probit model is selected. Therefore, FCSL_i_ can be modeled by a threshold model with a cumulative probit link for r = 1, 2 as follows:


(1) $$\begin{array}{l} \;p\left(FCSL_{i}\leq r\right)=P\left(D_{i}\leq \theta_{r}\right)=\\ P\left(\eta_{i}\;+\epsilon_{i}\;\leq \;\theta_{r}\right)=P\left(\epsilon_{i}\leq \;\theta_{r}-\eta_{i}\right)=\;F\left(\theta_{r}-\eta_{i}\;\right)\\ \;=>p\left(FCSL_{i}\leq r\right)=P\left(\epsilon_{i}\;\leq \;\theta_{r}-\eta_{i}\;\right) \end{array}$$

Similarly, Equation 1 can be given by *p*(*FCSL*_*i*_ ≤ *r*) = *F*(θ_*r*_−η_*i*_),

where F is the distribution function of the error variable ϵ_*i*_ of *D*_*i*_, by assuming that errors are Gaussian, i.e., ϵ_*i*_ ~ N(0, 1), leading to a cumulative probit link. However, the logit link also bring a closer result. The threshold model and cumulative probit link with an additive predictor extended to consider spatial effect, called a geo-additive mixed model (53). The geo-additive predictor given by the mixed model can help to detect non-linear covariates effect, structured spatial and random effect. Literature also indicated that the logit link can be an alternative, but the result obtained from cumulative logit and probit models for binary response (extended to ordinal) is usually quite close to each other (64, 65).

The geo-additive mixed model with empirical Bayes estimation is used to estimate an ordinal response predictor (η) to assess the zone-level structured and unstructured spatial distribution and the possible non-linear effects of covariates [see, (49, 66)] given by


(2) $$\begin{array}{l} \eta ={\text{X}}^{\text{T}}\gamma +f_{1}\left(z_{1}\right)+\ldots +f_{p}\left(z_{p}\right)+f_{unstr}\left(s\right)+f_{str}\left(s\right),\ldots \end{array}$$

where **X** = *x*_1_, …, *x*_*q*_ are categorical covariates of n × p matrix modeled by a linear function; **γ** = q × 1 vector of linear effects, **Z** = *z*_1_, …, *z*_*p*_ are continuous covariates of n × p matrix modeled by a non-linear function *f*_1_, …, *f*_*p*_, where *f*_*j*_(*z*_1*j*_), …, *f*_*j*_(*z*_*nj*_) = *f*_*j*_ = *Z*_*j*_β_*j*_.

Predictor model Equation (2) is an extended additive model by considering the spatial effects at an administrative zone level, and it can be split into strong correlation effect **f**_**str**_ (smooth, structured) and unstructured effect **f**_**unstr**_ (local correlation accounts for unobserved locally varying covariates). A rationale behind this split is that a spatial effect is usually a surrogate of many unobserved influential factors, some of them may obey a strong spatial structure, and others may be present only locally (49). The spatial information is given by zone map “connected geographical areas” and zone coordinates. We want to test the hypothesis that neighboring zones are more likely in the food security level than any other zones.

The spatial modeling is conducted by using two neighboring methods. The spatial contingency is fitted by *Markov random field* (MRF) using zones neighborhood map (50), while spatial distance is fitted by the k(=4)-nearest neighbors weighting method using a tensor product of zone longitude and latitude. However, the zone-level unstructured spatial effect is fitted by the Gaussian random effect. For spatial effects, the precision matrix is given by an adjacency matrix instead of an inverse correlation matrix (kriging) (40, 41, 64, 67, 68).

*Neighboring matrix:* For the given geographical map, zones are defined as neighbors if they share a common boundary and are assumed to be more likely in the food security level than any other zones, and the spatial effect is modeled by MRF (50). However, for zones longitude–latitude spatial distance neighboring, the spatial effect is fitted by a two-dimensional first-order random walk prior using a tensor product of zone coordinates (longitude and latitude) (69), and four nearest neighbors are taken (69), which is a type of isotropic correlation that does not depend on the direction of neighbors (48, 50, 53, 69–71). The neighborhood matrix for spatial contingency, with a value of −1 for zones sharing a common boundary otherwise, 0; and the spatial distance (four neighbors) are obtained from the Ethiopian shapefile map “Eth_Zone_2013” and the “bnd” object “mp_zn” is created directly using functions from the R package BayesX. A two-dimensional surface smoother called stationary Gaussian random fields (GRFs) can be used as an alternative (52). However, MRF's and P-splines are preferable to GRFs from a computational point of view, because their posterior precision matrices are band matrices or can be transformed into a band matrix-like structure, which speeds up the computation (49). The two-dimensional P-splines with 3 degrees and 20 equally spaced knots are applied for estimation.

The non-linear effects of continuous covariates are modeled through Bayesian versions of penalized splines (P-splines). The non-linear effects, *f*_1_, …, *f*_*p*_, are modeled non-parametrically using P-splines with a second-order random walk penalty. The P-spline used is a linear combination of B-spline base function *B*_*j*_(*x*_*j*_) estimated by $f\left(x_{j}\right)=\overset{S_{j}}{\underset{s=1}{\sum }}\beta_{j}B_{j}\left(x_{j}\right)$ (50, 72). Each of the base functions is constructed as a combination of piecewise polynomials of degree 3 defined on a set of equally spaced knots *x*_*j, min*_ = ζ_*j*, 0_ < ζ_*j*, 1_ < … < ζ_*j, k*−1_ < ζ_*j, k*_ = *x*_*j, max*_ within the domain of *x*_*j*_. We used 20 knots because the previous literature suggested 20 knots as a moderate larger number of knots (49, 64, 69, 72). However, the choice for number of knots should get critical attention in regression spline, because a spline with smaller number of knots may not sufficiently flexible to capture the data variability and for a spline with a significant number of knots (large variance τ^2^). The estimated curves may tend to over fit the data, and as a result, excessively rough functions are obtained. The number of knots is directly controlled by the size of the variance τ^2^. Large variance τ^2^ is equivalent to large number of knots, and small variance τ^2^ is equivalent to fewer number of knots. Hence, the complex problem of choosing optimal knots is replaced by the much simpler problem of finding an optimal smoothing parameter “variance τ^2^” (49, 53, 64, 72, 73).

The probability of a specific category r for the given effect is the area under the density between θ_*r*−1_ *and θ*_*r*_ thresholds. The influence of the effects is determined by the direction and magnitude of a shift on the distribution (64, 65).

*Priors:* Diffuse prior *p*(γ)∝*const* for fixed effect, Gaussian priors for i.i.d. random effects, MRF prior for spatial effect. A prior for a function *f*_*j*_ is based on specifying a suitable design matrix **Z**_**j**_ and a prior distribution for the vector **β**_**j**_ unknown parameters (40, 49). Here, the prior for the first- or second-order random walks for the regression coefficients is used and defined by β_*js*_ = β_*j, s*−1_+*u*_*js*_ and β_*js*_ = 2β_*j, s*−1_−β_*j, s*−2_+*u*_*js*_, respectively; with Gaussian errors $u_{js}\sim N\left(0,\;\tau^{2}\right)$ and diffuse priors *p*(β_*j*1_)∝*cons* or *p*(β_*j*1_)∝*cons* and *p*(β_*j*2_)∝*cons* for the initial value (47, 49, 64).

###  Method of estimation and model comparison

The inference is based on Empirical Bayesian, since REML estimators of variance components are less biased compared to the full Bayesian and the convergence problem is a challenge in MCMC. Here, priors are specified for the regression parameters and the hyperparameters (the smoothing parameters) are treated as fixed and estimated in advance from the data rather than from the prior, since the prior information is not sufficient on country wide household's food insecurity levels, specially specific to the corrected FCSL. The mixed model methodology estimates the regression coefficients using penalized likelihood and the variance parameter **τ**^**2**^ using restricted maximum likelihood (47, 49, 50, 64).

The R software interface for BayesX called R2BayesX package (41, 64, 67) is used for analysis. The R2BayesX is introduced by Umlauf et al. (41) which adds extensive graphics capabilities. The formula interface of the BayesX model used a variety of different smoothness priors depending on the type of covariate and the prior assumptions on smoothness. In this study, a continuous covariate is modeled using random walk priors (40) with Bayesian P-splines (69). The spatial effects are captured by Markov random field priors (74) and a tensor product of two-dimensional P-splines (71). Moreover, the prior of the regression coefficients has been estimated with a constant variance derived from the data (49). Model compression is achieved using the generalized cross-validation (GCV). A model with the smallest GCV value is selected as the best fit to the data (40, 49).

## Result

Descriptive statistics of the households' characteristics across the levels of food insecurity are shown in Table 1. This result indicated that 25% of the sampled households are food insecure and 27.08% are vulnerable to food insecurity. Chronologically, the insecurity decreased from 28 % to 23% and vulnerability also shows a slight decrement from 28% to 27% from year 2012 to 2016. The global Moran's I spatial autocorrelation indicated that the food insecurity level is significantly clustered across zones.

**Table 1:** Descriptive statistics of the households” characteristics across the levels of food insecurity (three times replicatly surveyed on *n* = 3,835 households).

**State of food security levels**	**Food insecure (0–35.5)**	**Vulnerable (35.5–49)**	**Food secure (> 49)**
FCS	25%	27.1%	47.9%
Rural vs. urban	26.1% vs. 16.6%	27.7% vs. 22.8%	46.2% vs. 60.6%
Read and write (no vs. yes)	29.5% vs. 18.6%	29.6% vs. 23.4%	40.9% vs. 58%
Employed (no vs. yes)	26% vs. 16%	27.7% vs. 21.1%	46.3% vs. 62.9%
Land own (no vs. yes)	22.7% vs. 25.3%	25.7% vs. 27.3%	51.6% vs. 47.4%
Shock (no vs. yes)	24.9% vs. 25.1%	26.6% vs. 27.5%	48.5% vs. 47.4%
Sex of household head (female vs. male)	29.9% vs. 23.3%	29.6% vs. 26.2%	40.5% vs. 50.5%
Fertilizer (no vs. yes)	24% vs. 25.8%	27.1% vs. 27%	48.9% vs. 47.2%
Farm types (cropping vs. livestock vs. both)	35.6% vs. 18.6% vs. 24.3%	29.1% vs. 23.8% vs. 27.1%	35.3% vs. 57.6% vs. 48.6%
Year 2012	28%	28%	44%
Year 2014	24%	27%	49%
Year 2016	23%	27%	50%
**Regions**			
Afar	21.1%	25.6%	53.2%
Amhara	24.8%	30.4%	44.8%
Benshagul–Gumuz	24.4%	33.3%	42.3%
Diredwa	14.1%	22.6%	63.3%
Gambelia	18.5%	24.6%	56.9%
Harari	20%	26.1%	53.9%
Oromia	14.6%	23.1%	62.2%
SNNP	39.2%	26.8%	34%
Somali	12.4%	23.9%	63.7%
Tigray	26.2%	31.2%	42.6%

Moran's I spatial autocorrelation result of ArcGIS: Moran's Index =1.2, and p = 0.0000.

Larger difference on the prevalence of food insecurity levels across regions was observed; specifically, the food insecurity of Somali and SNNP regions is the cold spot (12.4%) and as hot spot (39.2%), respectively, and more vulnerable households are residing in the Benshagul-Gumuz region (33.3%) and the lowest proportion is residing in the Diredwa region (22.6%). The spatial dependency is observed on longitudinal average of the food insecurity levels (see Figure 1B), and this result suggests that most northern and south-western parts of Ethiopia are food insecure, and most of the central and western part of Ethiopia are vulnerable to food insecurity.

Food insecurity and vulnerability are higher among rural (26.1% and 27.7%), female headed (29.9% and 29.6%), and illiterate who cannot read and write (29.5% and 29.6%) households, as opposed to their counterparts in urban areas (16.6% and 22.8%), male headed households (23.3% and 26.2%), and those can read and write (18.6% and 23.4%), respectively.

On the other hand, households that were more food insecure and vulnerable tended to face shock/s (25.1% and 27.5%), small land ownership (25.3%and 27.3%), and an unemployed head (26% and 27.7%) compared to their counterpart. Conversely, households not facing shocks (24.9% and 26.6%), no owned land (22.7% and 25.7%), having employed head (16% and 21.1%) exhibited lower food insecure and vulnerable levels. The proportion of food insecure and vulnerable households engaged in crop farming only was greater (35.6% and 29.1%) compared to those involved in livestock farming (18.6% and 23.8%) or both (24.3% and 27.1%), respectively. Similarly, the proportion of food insecure households using fertilizer was smaller (23.99%) compared to the households not using (25.80%).

### Empirical Bayes geo-additive mixed model result

**Figure 2:**
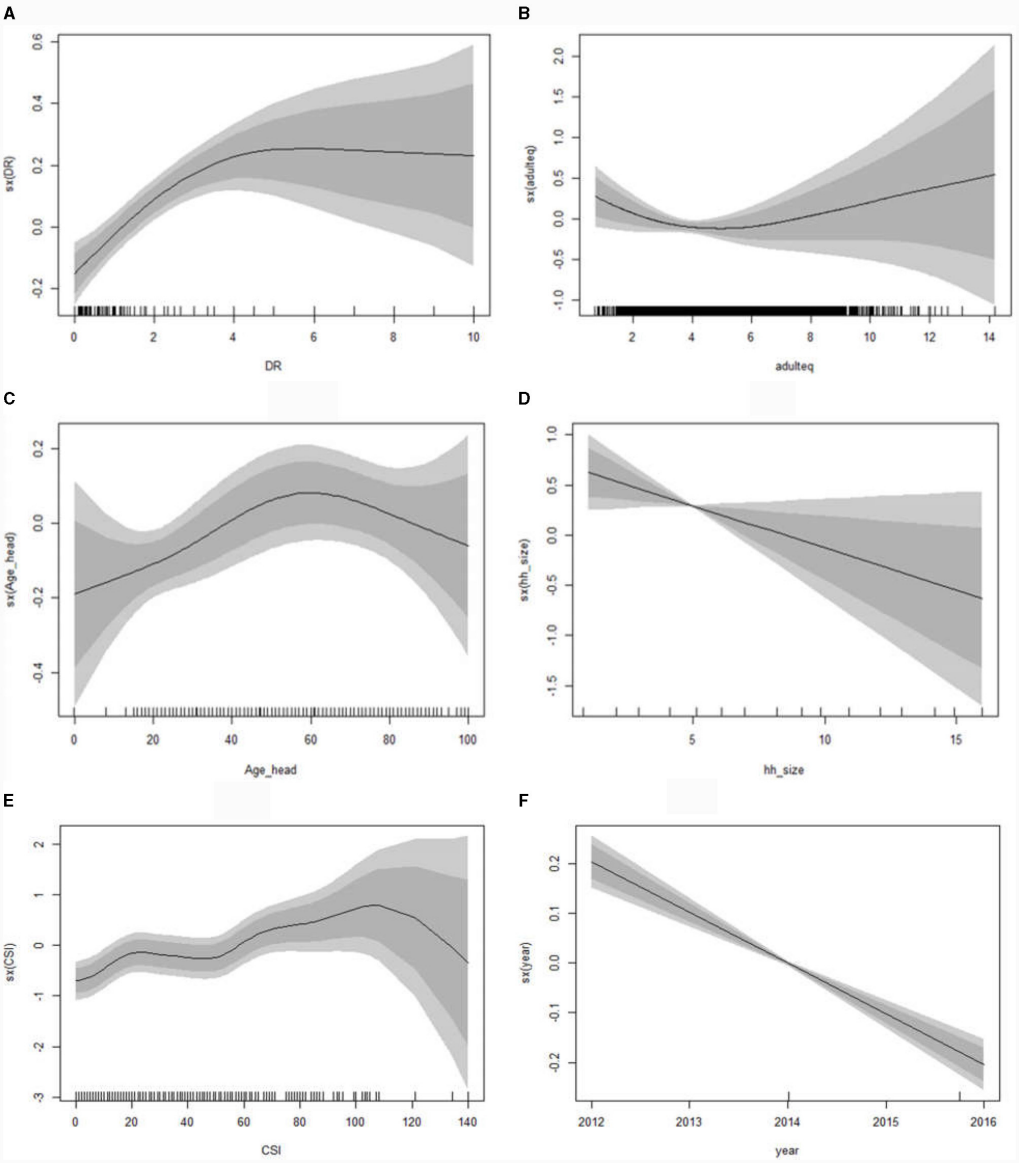
Posterior mode estimates for the non-linear effects; dependency ratio **(A)**, adult equivalence **(B)**, age of household head **(C)**, household size **(D)**, Coping Strategy Index **(E)**, and survey year **(F)** (with 95% CI) on levels of food insecurity using a geo-additive model.

**Figure 3:**
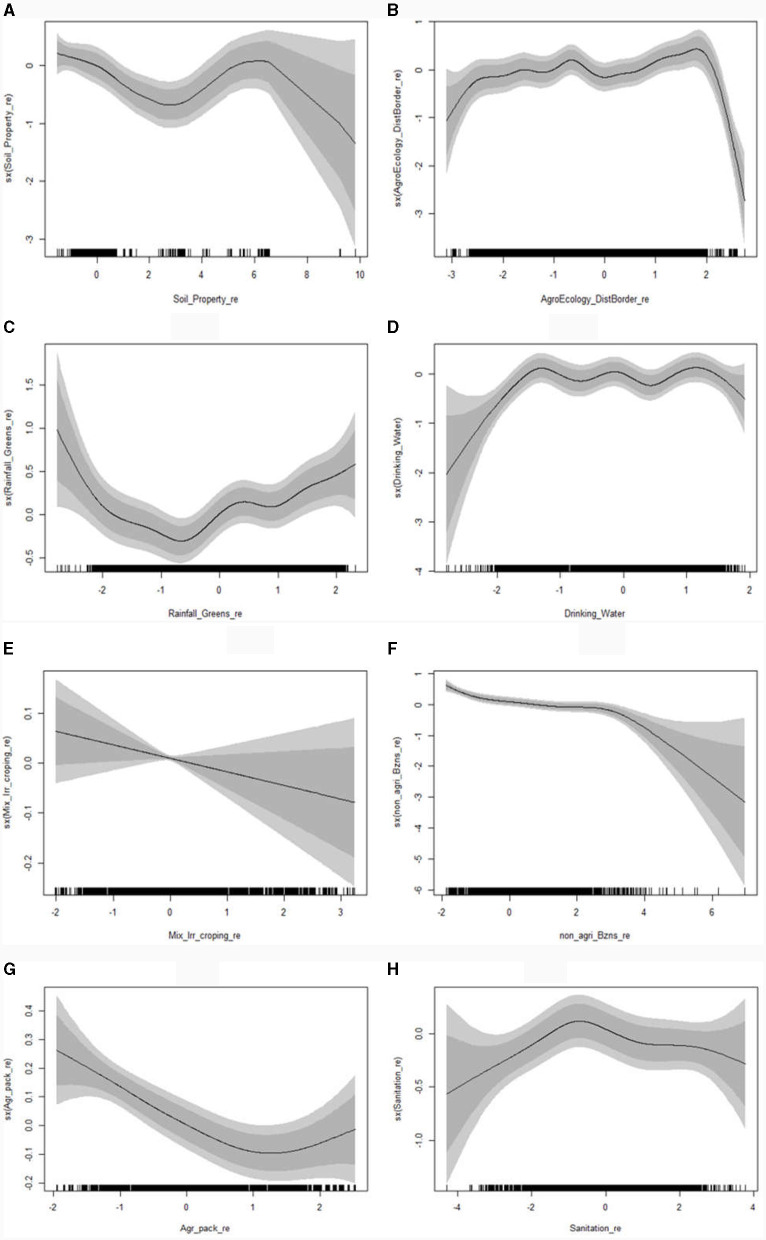
Posterior mode estimates for the non-linear effects; soil property related **(A)**, agro-ecological and distance from border related **(B)**, rainfall and greenness related **(C)**, drinking water **(D)**, irrigation, mixed cropping and related **(E)**, non-agricultural business related **(F)**, agricultural package related **(G)**, and sanitation related **(H)** (with 95% CI) on levels of food insecurity using geo-additive model.

**Figure 4:**
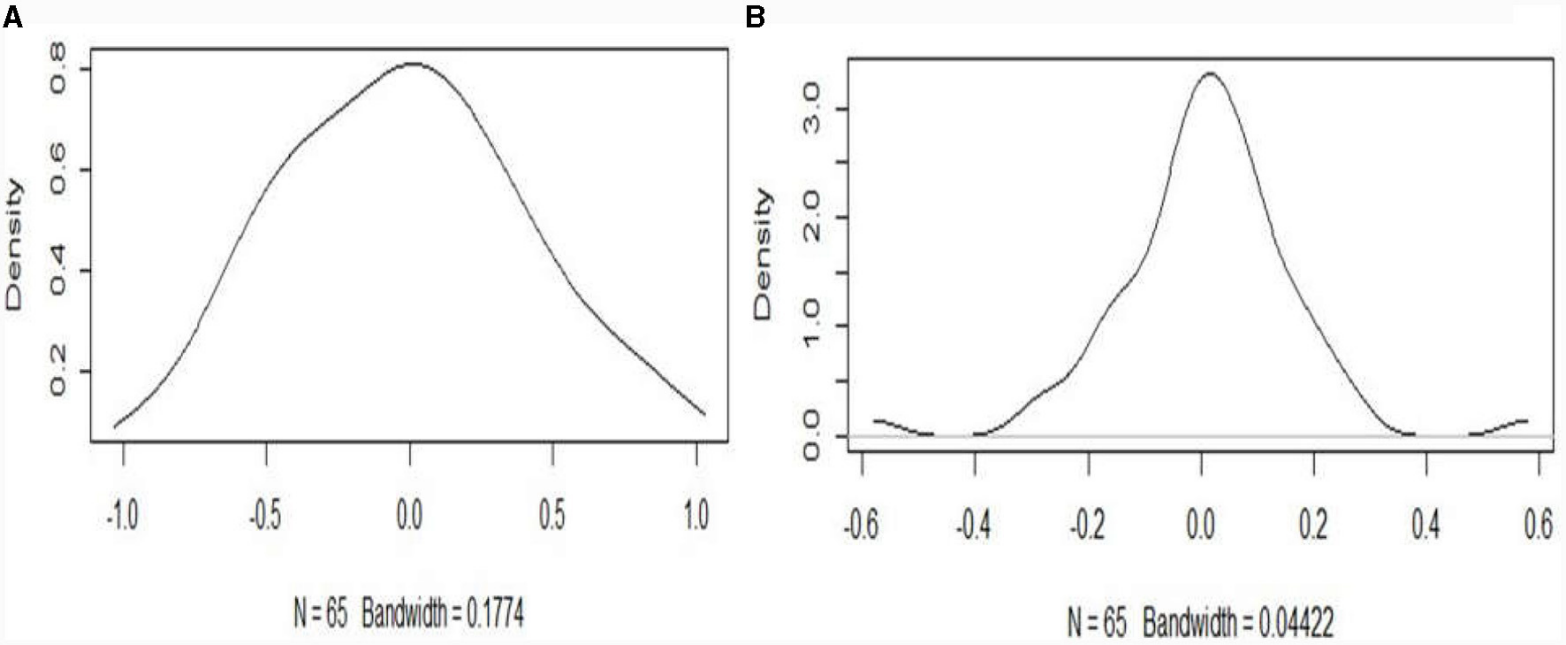
Kernel for structured **(A)** and unstructured **(B)** spatial covariate effect.

From extending the result of an additive model, based on the observed spatial clustering in Figure 1B) and global Moran's I spatial autocorrelation suggestion in Table 1, the spatial effect modeled by Markov random filed (MRF Model) introduces smoothness and effect change, yielding a smaller GCV of 1.8221 (see Figures 2, 3, and Table 2) compared to the additive model with GCV of 1.9550 (Supplementary Figures S1, S2, and Supplementary Table S1). Additionally, the spatial covariate modeled by tensor product shows a GCV of 1.8223 for 8 knots and a GCV of 1.8224 for 20 knots. Moreover, the kernel plot of the MRF model in Figure 4 indicated that the spatial correlation effect range is wider (−1 to 1) and flatter Gaussian distribution with higher variance estimate of the spatial effect of 0.6104 (i.e., 90.04% from the total variation is explained by the spatial effect), compared to the random effects with a range from −0.4 to 0.4 and a variance of 0.0675. This indicated that model fit improvement by including random effects that account for the unobserved spatial heterogeneity of the zones in Ethiopia, is relatively low, hence, the focus should be on spatially structured effects.

**Table 2:** Posterior model estimates: geo-additive model for spatial correlation modeled by Markov random field and uncorrelated effect by random effect.

**Fixed effects estimation results: parametric coefficients**	**Estimate**	**Std. error**	***t-*value**	**Pr(>|t|)**
θ_1_	−0.3789	0.2185	−1.7338	0.0830(.)
θ_2_	0.4742	0.2186	2.1694	0.0301(^*^)
Urban vs. Rural	−0.3883	0.0440	−8.8324	0.0000(^***^)
Read and Write (Yes/No)	−0.2378	0.0262	−9.0825	0.0000(^***^)
Shock (Yes/No)	0.0683	0.0239	2.8600	0.0042(^**^)
Fertilizer (Yes/No)	−0.1324	0.0349	−3.7953	0.0001(^***^)
Employed (Yes/No)	−0.2054	0.0427	−4.8115	0.0000(^***^)
Health problem (Yes/No)	0.0281	0.0271	1.0387	0.2990
Small size land ownership (Yes/No)	0.1566	0.0424	3.6947	0.0002(^***^)
Farm Type: (Livestock/Cropping)	−0.3231	0.0672	4.8057	0.0000(^***^)
Farm Type: (Both farms/Cropping)	−0.1923	0.0407	−4.7251	0.0000(^***^)
**Smooth terms**	**Variance**	**Smooth Par**.	**df**	**Stopped**
Adult equivalence	0.0009	1126.9300	3.6938	0
Age of household head	0.0001	6961	2.2735	0
Agricultural package related	0.0001	10027.9000	2.5430	0
Agro-ecological and distance from border related	0.1081	9.2529	10.9175	0
Coping strategy index	0.0140	71.2708	5.5753	0
Dependency ratio	0.0001	6878.3100	2.3204	0
Drinking water	0.0216	46.2039	7.6259	0
Household size	0.0000	168901	1.0385	1
Irrigation, mixed cropping and related	0.0000	28818900	1.0015	1
Non-agricultural business related	0.0049	202.4790	4.7566	0
Rainfall and greenness related	0.0087	115.2580	6.5107	0
Sanitation related	0.0017	592.6620	3.9997	0
Soil property related	0.0102	97.7056	4.2269	0
Year	0.0000	4312010	1.0468	1
Structured spatial effect (MRF)	0.6104	1.6383	40.3100	0
Unstructured spatial effect	0.0675	14.8126	19.2056	0

Signif. codes: 0 “^***^” 0.001 “^**^” 0.01 “^*^” 0.05 “.” 0.1 “1”. N = 11,505, df = 128.046, AIC = 21694.4, BIC = 22635.6, logLik = –10719.15, GCV = 1.8221.

**Figure 5:**
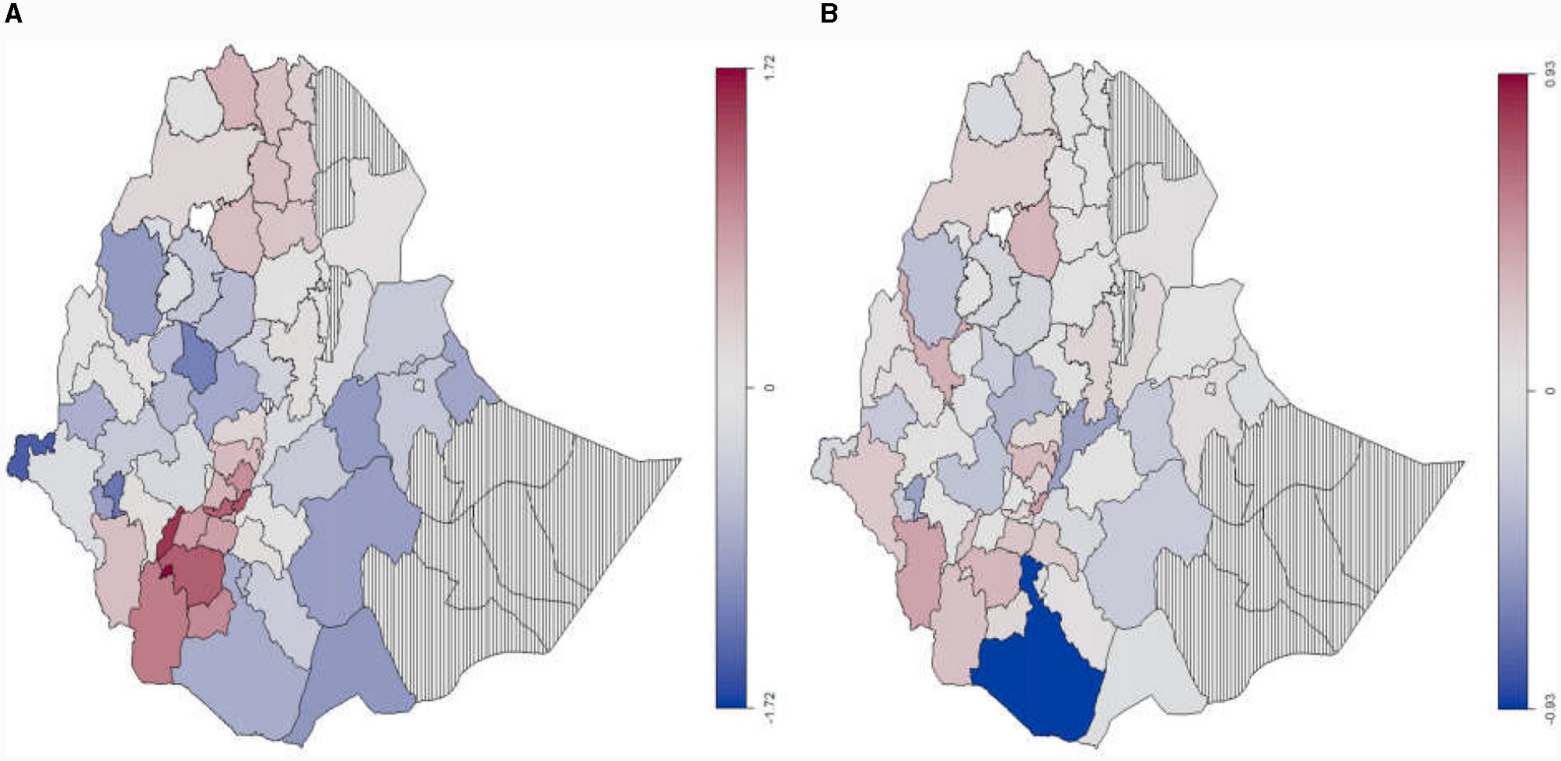
A visual representation of the posterior means for structured spatial effect fitted by Markov random field prior **(A)** and unstructured spatial effect fitted by Gaussian prior random effect **(B)** for levels of food insecurity (zones with vertical lines represent areas with no data).

The MRF model in Figure 5 for spatially correlated map effect (a) indicated that the majority of southern-west and northern areas of Ethiopia have higher food insecurity (red colored). This result also reveals that the greater number of zones in the south-east and north-west areas of the country are vulnerable to food security (whiten colored).

The results in Table 2, detailing the posterior mode estimates of the linear effects, reveal that factors such as illiterate or educated (β = – 0.2378^***^), employment (β = −0.2054^***^), residing in urban areas rather than rural (β = −0.3883^***^), fertilizer usage (β = −0.1324^***^), and farm types either livestock (β = −0.3231^***^) or a combination of livestock and cropping (β = −0.1923^***^) exhibit significant reducing effects. Conversely, the shock occurrence (β = 0.0683^**^) and small land ownerships (β = 0.1566^***^) exhibit a significant increasing effect on household food insecurity and vulnerability.

The posterior mode estimates of the non-linear effects in Figure 2 indicate that the household's food security level showed a positive linear significant progress over years (Figure 2F). It also presented that household size has a significant linear positive effect on a family's food security level (Figure 2D). It also depicted that the household's dependency ratio and coping strategy index have significant non-linear effects on the household's food (Figures 2A, E, respectively), whereas the effects of adult equivalence and household's head age are non-linear (Figures 2B, C, respectively), even if there is no sufficient evidence to support the statistical significant at 95% confidence level. However, the result of household age revealed the real phenomena of Ethiopian early marriage problem, wherein food insecurity increases for younger household heads and then declines for older household heads. It is known that 80% of the population resides in rural areas, where marriage is commonplace. Newly established families, with some wealth gained from their parents, struggle to fulfill their basic needs, including food consumption, renting a new house, investing assets, and supporting new born. This issue increases the food insecurity among young household heads. However, this state tend to improve after several years of hard work at their older age.

Figure 3 also showed that the component of soil property; component of agro-ecology, distance from border and related; a component of rainfall, greenness and related; drinking water; component of non-agricultural business and related; component of agricultural package and related; and sanitation related, exhibit non-linear effects on household's food. With the exception of sanitation-related factors, all other components have a significant effect on food outcomes (Figures 3A, B, C, D, F, G, respectively). This aspect also indicates that the component of irrigation, mixed crop, and related has a linear effect, even if there is no sufficient evidence to support the significant at 95% confidence level (Figure 3E).

## Discussion

The descriptive statistics indicates higher prevalence of food insecurity (25%) and vulnerability (27.08%), implying that more than half of the population had a problem of feeding (in hanger), even if positive progress in time has been observed in reducing the proportion insecure and vulnerable households. In-line with this result, the 2022 report of State of Food Security and Nutrition in the World (SOFI) showed that 56% of the total population affected by moderate or severe food insecurity between 2019 and 2021 (23), and 20 million people are in need of food by 2023 (99). Although severity of food insecurity in the country is also reported by several studies (22, 28, 29, 31, 75–77), the vulnerability is not assessed as such. In the meantime, as the literature supports, improved food aid and safety nets programs have to be strengthened to control chronic food insecurity (23, 74, 77).

This study aims to find out the hot spot zones of food insecurity and vulnerability zones based on the spatial distribution of the household's food insecurity levels following the investigation of the contributing factors to each levels of food insecurity from geographic, environmental, and socioeconomic variables for the purpose of mitigation for each specific area/zone. Comparing to previous study conducted in developing countries (18, 54, 76–79) and Ethiopia (22, 29, 31, 34, 80, 81), this study measures the household's food insecurity levels by corrected FCSL using cut-points, which consider that local factors lead to divers feeding pattern, analyze the non-linear effect of geographic, environmental, and socioeconomic factors, and identify causing determinants to each levels of food insecurity to implement the area-specific mitigation for hot spot zones of food insecurity and vulnerability.

Though several researches assessed the determinants of Ethiopian household food insecurity and supported the associated factors obtained in this study (31, 34, 80, 81), only few researches consider the spatial pattern in Ethiopia (22, 82, 83) with better literature on developing country (18, 84–90). However, none of literature labeled as food insecure, vulnerable, and secured based on cut-points corrected for feeding cultural difference and provided the determinants specific to the hot spot zones of food insecurity and vulnerability.

Therefore, to assess the geographic distribution and find out the effects (linear and non-linear) of factors on household's food insecurity levels, the additive model is extended for the spatial effect using MRF and tensor product and unobserved effect by Gaussian. Using GCV, the MRF geo-additive mixed model is selected as a best fit. The advantage of introducing spatial effects to an additive model is reported by Kammann and Wand (52), and specifically, this model is elaborated by Fahrmeir et al. (49). However, in the MRF model, the implication of the higher variance estimate of the spatial effect of 0.6104, i.e., 90.04% of the total variation, is explained by the spatially structured effect compared to the unstructured effects, indicating that the model fit improvement by including random effects that account for the unobserved spatial heterogeneity of the zones in Ethiopia is relatively low. Hence, spatially structured effects on household's food security levels should be focused. A similar suggestion had been reported by previous researches (41, 47).

The spatially structured effect result from the MRF model reveals that the majority of the south-west and northern part of Ethiopian zones have higher food insecurity and south-east and western part of Ethiopian zones are more food secured, especially Nuer zone has better food security. A similar result of the zone-level spatial effect has been suggested by Dessie et al. (22). Specifically, North Tigray, Western Tigray, Central Tigray, Southern Tigray, Wag Himra, South Gonder, North wollo, South Omo, Basketo, Gamo Gofa, Segen Peoples', Konta, Dawro, Wolayita, Gurage, Selti, Hadiya, Alaba, KT exhibit higher food insecurity. A similar result for smoothness due to considering the zone-level spatial effect is reported by Dessie et al. (22), and other studies also indicated the presence of regional variation in food security (39, 91–93). The distinguished finding from this result is that the greater number of zones in the South-East and North-West areas of the country re vulnerable to food insecurity. Actually, the unstructured spatial effect also agree on some of these results, which may be due to similarity of locally (zone level) influencing factors of vulnerability.

The posterior mode estimates of the linear effects revealed that an increase in urbanization, education, and job/employment decreases the higher food insecurity levels (food insecurity and vulnerability). The urbanization effect shows a higher magnitude in reducing higher levels of food insecurity compared to other effects. The literature also strongly supports the influence of urbanization on reducing food insecurity levels, meaning that rural households are more food insecure than urban households (22, 29, 36, 94, 95). The positive effect of education to mitigate food insecurity is indicated by previous researchers (28, 31, 32, 76, 78, 80, 91, 96, 97), and similarly, the contribution of employment to reduce food insecurity is also supported by previous researchers (31, 54, 77, 78, 80, 95, 98). Food insecurity tends to increase for younger household heads and subsequently declines for older household heads. Since 80% of the population resides in rural area where early marriage is commonplace, younger households struggle with establishing a new house, investing assets, and supporting their new born. This state increases the food insecurity during the early years of young household heads, but this situation tends to improve after several years of hard work at their older age. Therefore, early marriage poses a higher risk of food insecurity for younger household heads compared to older household heads (22, 31, 54, 80).

Farming livestock or both livestock and crop has a significant effect on reducing higher levels of food insecurity (14, 31, 54, 75, 76, 80, 95, 99). Fertilizer usage reduces the higher food insecurity levels. This result coincides with the previous study results (22, 31, 32, 75, 100).

The shock occurrence increases the higher food insecurity levels. The severe effect of shock is reported by previous studies (22, 29, 36, 94, 101, 102). Households with small land sizes are strongly positively associated with the higher levels of food insecurity. The negative effect of small land size owned households on the reduction of food insecurity is supported by previous studies (31, 32, 75, 95, 99, 102).

The posterior mode estimates of the non-linear effects indicate that the household's food security levels showed a positive linear significant progress over years. Predominantly, the significant reduction of food insecure and vulnerable households in the country has been supported by the literature (29, 103) and distinguished from the aggravation of recent years global food insecurity prevalence (3, 15). The result also reveals that the probability of being food insecure increase with the household's dependency ratio; however, at a higher level of dependency ratio (> 4), the effect keeps constant. Specifically, when a dependency ratio in a household moves from 0 to 1, the level of food insecurity turns from a negative to a positive scale, implying that the Ethiopian household's food security does not hold any dependent individual in the family. This result is supported by a similar study conducted by previous researchers (22, 29, 104–106).

The household size has a significant linear negative effect on a family's probability of being in higher levels of food insecurity because in Ethiopia agriculture is human labor based and having higher human power can help in gaining much production from farming crops and livestock in rural areas to secure family's food intake. This result is in contrast with previous studies (6, 75, 97); however, the importance of human power resources for productivity in food security is well explained by previous studies (1, 36). The strength of coping strategy shows a monotonic increasing association with the higher levels of food insecurity up to 100, but beyond that point, food insecurity steps down. Hence, the applied coping strategy depends on the severity of food insecurity. Previous research has identified that different coping strategies are applied based on the magnitude of food shortage (29, 107).

The posterior mode estimates of the non-linear effects indicated that as soil property increases, the probability of a household in higher levels of food insecurity declines at the lower level (0 to 3). However, at the middle score ranging from 3 to 6, the effect is insignificant even though the probability appears to increase again. This increase is disrupted and declines at higher scores, which may be due to soil having higher (an increased) nutrients. Oxygen can lead to higher production from farming crops and livestock. This result is supported by previous studies (80, 95).

At a higher score agro-ecological zone (>2), a decline in the higher levels of food insecurity is observed, whereas a linear increase in higher levels of food insecurity was observed up to −2, but from −2 to 2, the change was not visible. This aspect implies that the temperate (colder), central, and highland areas exhibit better food security or less probable to be in a higher levels of food insecurity. This result is supported by previous studies (6, 80, 92, 94). The increase in components of rainfall and greenness declines the higher levels of food insecurity at lower values (< 0), but it rises at higher values (0 to 2). Therefore, excessive rainfall and precipitation can reduce production rather a moderate score can help to reduce higher levels of food insecurity and helps to secure food. A similar result for the effect of rainfall and greenness on food insecurity is reported by previous studies.

The probability of the higher levels of food insecurity increases up to the drinking water score of −2, remains constant up to 1, but declines after reaching 1, which implies that having drinking water source >1 leads to a significant reduction in the household's probability of being at the higher levels of food insecurity. This result is supported by a previous study (102). The previous study also indicates that a component of irrigation, mixed crop, and related has a linear positive effect on reducing a household's probability of being at the higher levels of food insecurity. The contribution of irrigation, mixed cropping, and land conservation to the reduction of food insecurity is reported by previous studies (35, 97).

An increase in non-agricultural businesses reduced the probability of a household being at the higher levels of food insecurity. However, having more than three non-agricultural businesses leads to a higher food security. The effect of more income from the non-agricultural business is reported by many studies (31, 80, 84, 97). The probability of a household being at the higher levels of food insecurity declines as agricultural package usage increases up to 2, but using more than two agricultural package increases it and consider as wasting effort. The improvement obtained in food insecurity from agricultural package implementation is also reported by previous studies (80, 95). The probability of a household being at the higher levels of food insecurity increases when sanitation is negative (< 0). Conversely, for positive sanitation, the probability declines faster, implying that a household with higher sanitation (better solid waste disposal, bathing and toilet) has a higher probability of being food secure. This result is supported by previous studies (29, 34, 96).

In general, this study assessed the spatial heterogeneity of household food insecurity levels (18, 22) and identified the underling driving factors to the hot spot zones of food insecurity and vulnerability. Hence, the higher levels of food insecurity (insecure and vulnerable) can be reduced by working on urbanization (22, 29, 36, 94, 95), education (28, 31, 32, 76, 78, 80, 91, 96, 97), reduction in young household head by combating early marriage (22, 31, 54, 80), using fertilizer in cropping (22, 31, 32, 75, 100), creating jobs/employment (31, 54, 77, 78, 80, 95, 98), farming livestock or/and crop (14, 31, 54, 76, 80, 95) agricultural package related (80, 95), soil property related (80, 95), rainfall and greenness related (34, 80), irrigation and mixed cropping and related (34, 35, 97), agro-ecological and distance from border related (6, 22, 34, 80, 92, 94), household size (1, 36), non-agricultural business related (31, 80, 84, 97), sanitation related (29, 34, 96), and drinking water (102). Controlling factors, such as shocks (22, 29, 36, 94, 101, 102), dependency ratio (22, 29, 104–106), owned low land size literature (31, 32, 75, 95, 99, 102), and using better Coping Strategy Index, can help to reduce food insecurity and vulnerability.

## Strengths, limitations, and future work

Previous research on Ethiopian food security used cross-sectional data and focused on investigating the linear effects of factors on food insecurity. This paper has several strengths; this paper used the recommended locally adjusted cut-points of WFP's FCS, and identified that deriving factor leads to the hot- and cold spot food insecure areas further to uncover the general distribution of food insecurity levels across administrative zones of the country. We have used the available data that is older than 7 years, since recent data are not yet collected by the concerned body due to many problems faced in Ethiopia including political instability, war, and displacement. On the other side, even though a large sample size of 3835 households are taken repeatedly three times in year 2012, 2014, and 2016, the model does not converge and the longitudinal correlation due to repeated measures at the household level across space/zones is not considered due to the convergence problem. Actually, the model considered that only longitudinal evolution and correlation were converged but ignoring the spatial dependency does not address the objective and lower the relevance of the result. We have also faced convergence problem for full Bayesian estimation and even though the Empirical Bayes achieved convergence by stopping criterion for small variances less or equal to 1e-05. Therefore, as future work one can extend the work using sufficiently repeated measurements based on the panel data that will be released in the future and assess the evolutional nature of the household level food insecurity levels across space/area. Furthermore, there is also a need for further comprehensive researches that consider cultural disparities across nations, which affect the consumption pattern to fix a universal threshold for food insecurity levels cut-points or some robust estimate.

## Conclusion

The household's levels of food insecurity showed direct significant linear progress over the years. Hence, significant progressive reduction of food insecure and vulnerable households in the country has been observed.

The spatially smoothed additive model brings an advantage in identifying hot spot levels of food insecurity over the additive model by extracting effect of area/zone specific and structured determinants causing each level (insecure or vulnerable). The strong spatially correlated severe food insecurity across zones has been observed mainly in the northern and south-west parts of Ethiopia. However, the greater number of zones in the south-east and north-west areas of the country are spatially correlated vulnerable zones to food insecurity. Interventions are recommended to address spatial structure factors in these areas. Specifically, since the structured spatial effect explained the majority (90.04%) of the total spatial variation, the significant factors with large magnitude contributed a larger variance to the model. Thus, implementing policies, such as focusing on urbanization [since most rural areas are inadequately connected to urban due to low road infrastructure, farmers are out of modern inputs to achieve higher yields and sell or buy from the market (16), “targeting zone level urbanization is a key”], education, early marriage control, and job creation, can mitigate food insecurity and the vulnerability in hot spot zones. Moreover, considering the current conflict, frequent drought, population size, degraded land, and global shocks impact on the country, to meet future food security Agriculture's environmental footprint, must be controlled (108) by focusing on integrated farming for drought-resistant crops, such as climate-smart agriculture project (15) and insurance programs for livestock (15), through environmental and land conservation. Subsequently, applying the best coping strategies (relying on less preferred foods and limiting the variety of foods eaten) can help to reduce higher levels of food insecurity. This result can help policymakers and future researchers to apply and extend the study to overcome replicated data limitations and model convergence problems by adding the next (2nd) longitudinal data and high-performance computer for better understanding of the food insecurity levels dynamics in Ethiopia.

## Data availability statement

The 2011-2016 ESS data files and documentation are publicly available and can be found at: https://microdata.worldbank.org/index.php/catalog/2053, https://microdata.worldbank.org/index.php/catalog/2247 and https://microdata.worldbank.org/index.php/catalog/2783. Further inquiries can be directed to the corresponding author.

## Author contributions

HW: Conceptualization, Formal analysis, Methodology, Software, Writing—original draft. TZ: Conceptualization, Supervision, Writing—review & editing. AM: Conceptualization, Formal Analysis, Methodology, Supervision, Writing—review & editing. ZD: Data curation, Formal Analysis, Methodology, Supervision, Writing—review & editing.
