# Supplementary material for: The spatial effects of the household's food insecurity levels in Ethiopia: by ordinal geo-additive model
Source: Front Nutr. 2024 Feb 29;11:1330822. doi: 10.3389/fnut.2024.1330822 (PMC10939041; doi:10.3389/fnut.2024.1330822)
Supplement: Supplementary file 1 [file Data_Sheet_1.docx]

# Appendix

Table S1: Posterior mode estimates: Linear effect of categorical variables and non-linear effects of continuous covariates on FCSL from additive model.

| Parametric coefficients: | Estimate | Std.Error | t-value | Pr(>\|t\|) |
| --- | --- | --- | --- | --- |
| $\theta_{1}$ | -0.54 | 0.48 | -1.13 | 0.2605 |
| $\theta_{2}$ | 0.76 | 0.48 | 1.57 | 0.116 |
| Urban Vs Rural | -0.38 | 0.07 | -5.49 | <2e-16 *** |
| Read & Write (Yes/No) | -0.44 | 0.04 | -10.53 | <2e-16 *** |
| Shock (Yes/No) | 0.00 | 0.04 | 0.00 | 0.9962 |
| Fertilizer (Yes/No) | -0.08 | 0.05 | -1.54 | 0.1231 |
| Employed (Yes/No) | -0.37 | 0.07 | -5.26 | <2e-16 *** |
| Health problem (Yes/No) | 0.05 | 0.04 | 1.26 | 0.2086 |
| Small size land ownership (Yes/No) | 0.09 | 0.07 | 1.34 | 0.1808 |
| Farm Type:[Livestock/Cropping] | -0.52 | 0.11 | -4.86 | <2e-16 *** |
| Farm Type:[Both farms/Cropping] | -0.38 | 0.06 | -5.93 | <2e-16 *** |
| Signif. codes: 0 ‘***’ 0.001 ‘**’ 0.01 ‘*’ 0.05 ‘.’ 0.1 ‘ ’ 1 | | | | |
| Smooth terms: | Variance | Smooth Par. | df | Stopped |
| Adult equivalence | 0.00 | 346.50 | 3.90 | 0 |
| Age of household head | 0.00 | 1020.65 | 2.98 | 0 |
| Agricultural package related | 0.00 | 1794.80 | 3.11 | 0 |
| Agro-ecological & distance from border related | 0.02 | 40.32 | 7.01 | 0 |
| Copping Strategy Index | 0.13 | 7.44 | 7.28 | 0 |
| Dependency Ratio | 0.00 | 1268.90 | 2.76 | 0 |
| Drinking Water | 0.16 | 6.19 | 9.34 | 0 |
| Household size | 0.00 | 35630.60 | 1.06 | 1 |
| Irrigation, Mixed cropping &related | 0.00 | 3589.31 | 2.44 | 0 |
| Non-agricultural Business related | 0.01 | 200.67 | 3.75 | 0 |
| Rainfall & greens related | 0.09 | 11.02 | 9.38 | 0 |
| Sanitation related | 0.00 | 325.99 | 3.62 | 0 |
| Soil property related | 1.06 | 0.95 | 8.75 | 0 |
| Year | 0.00 | 50575.80 | 1.61 | 0 |
| Unstructured spatial effect | 0.0984 | 10.1577 | 36.1128 | 0 |
| N = 11505, df = 127.358, AIC = 21693.1, BIC = 22629.3 ,logLik = -10719.2, GCV = 1.82237 | | | | |


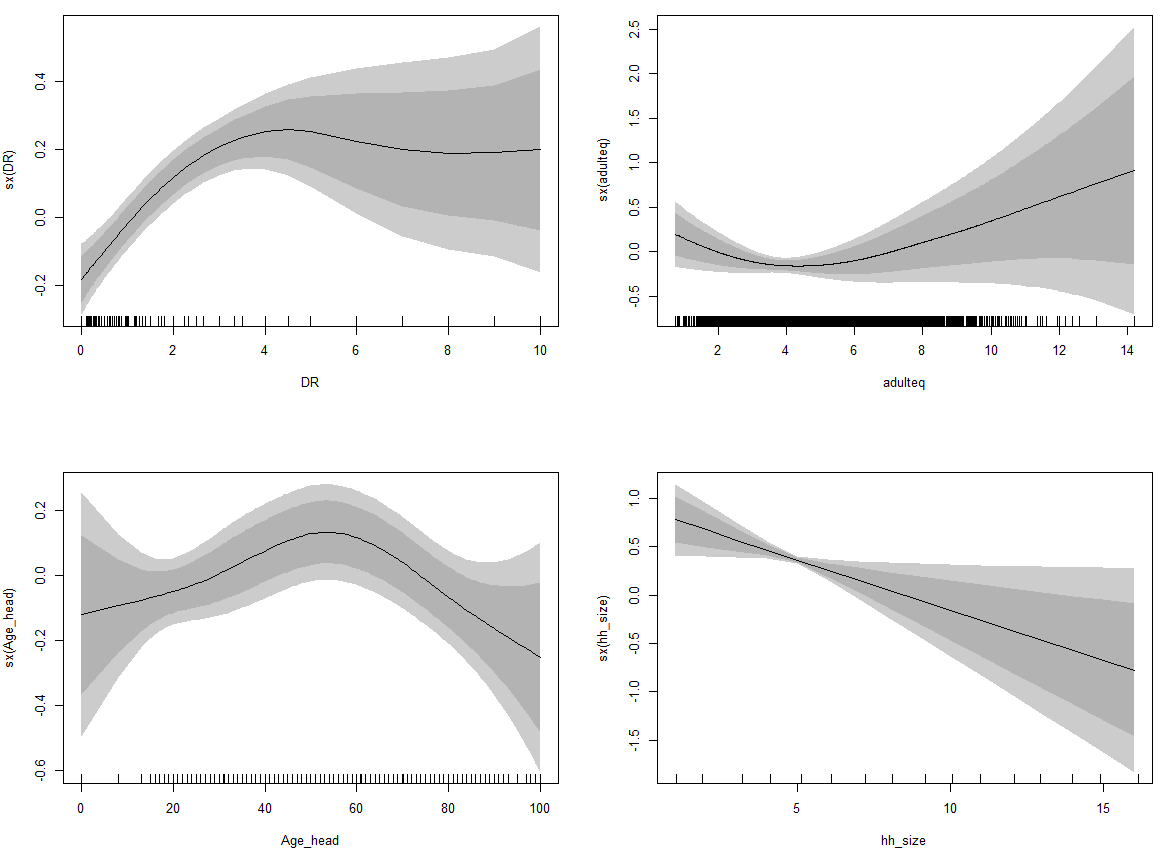


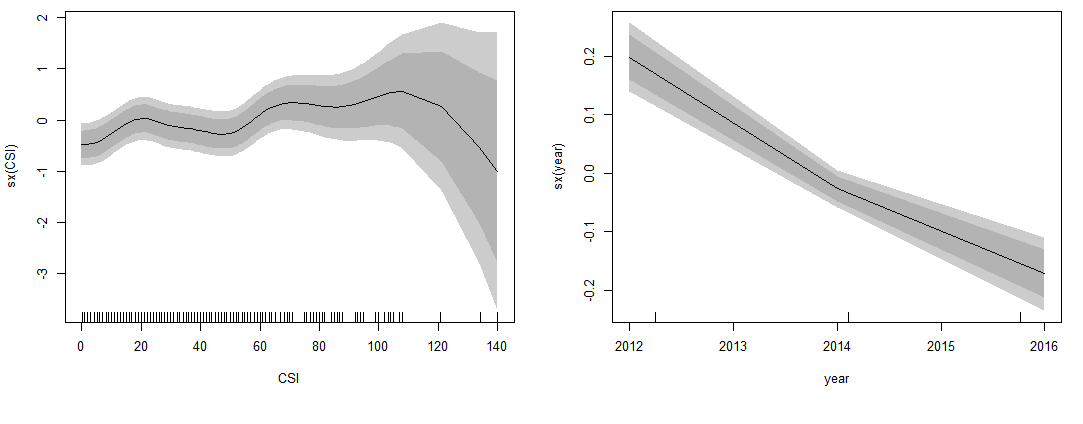


Figure S1: Non-linear effect on FCSL from additive model


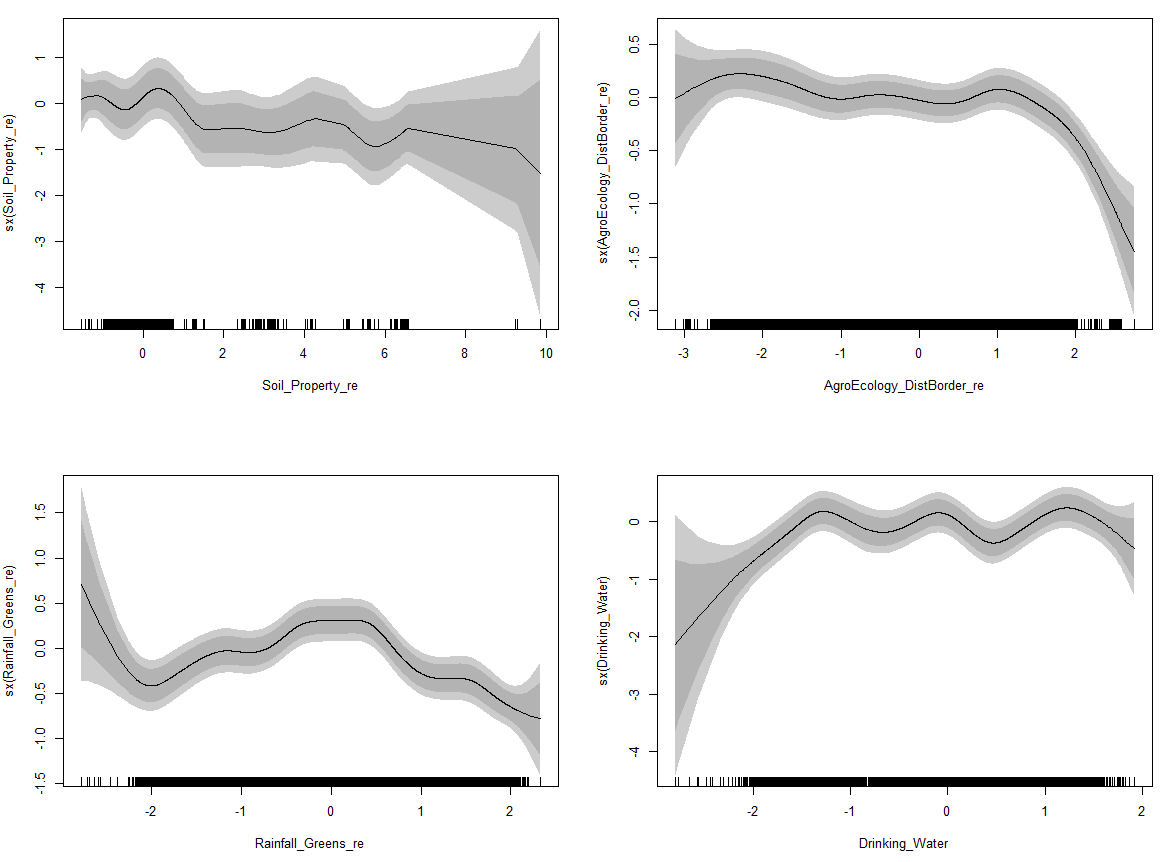


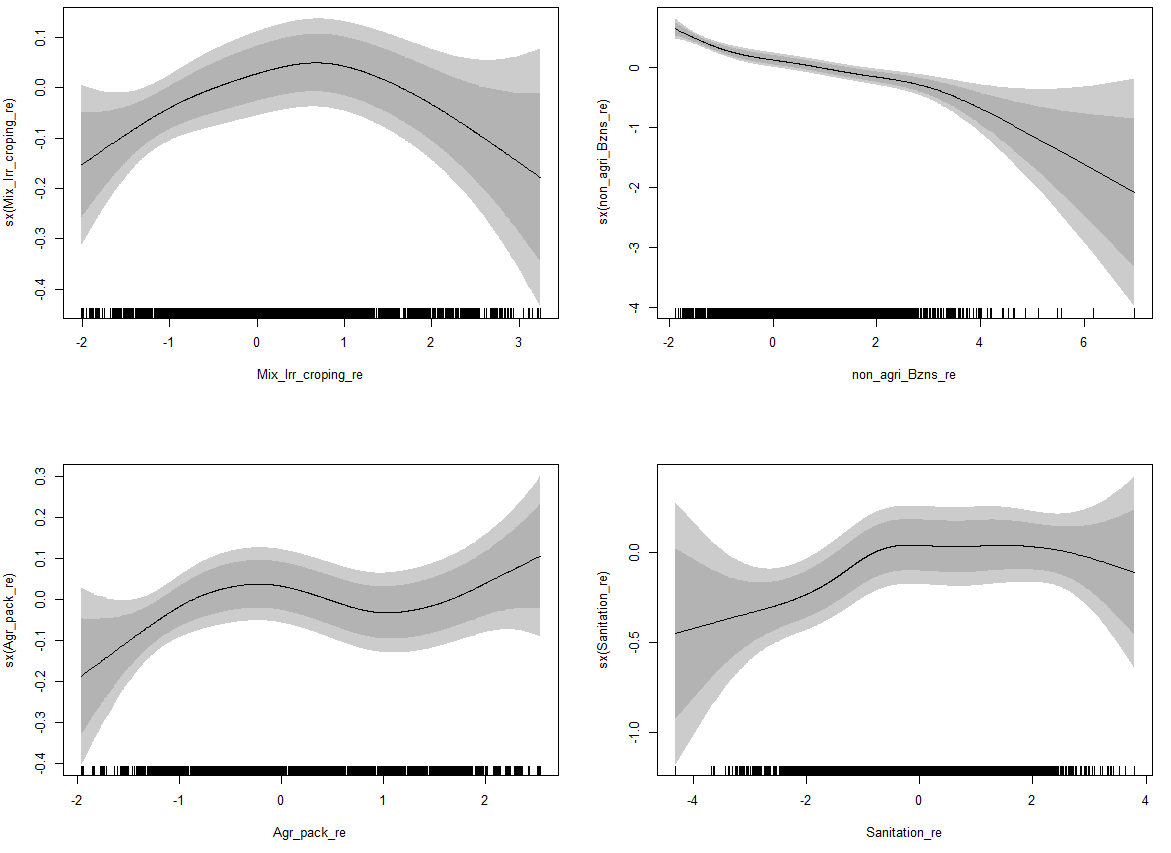


Figure S2: Non-linear effect on FCSL from additive model
